# Supplementary material for: Assessing how alcohol use patterns relate to obesity among American adolescents from rural and urban areas: Five years of pooled data
Source: PLoS One. 2024 Jun 27;19(6):e0305638. doi: 10.1371/journal.pone.0305638 (PMC11210796; doi:10.1371/journal.pone.0305638)
Supplement: S1 Table — (DOCX) [file pone.0305638.s001.docx]

**Supplementary Table 1. Descriptive characteristics of the study sample by Obese vs Non-Obese, National Survey on Drug Use and Health, Years 2015-2019, Adolescent sample (age 12-17; n = 39,489)**

|  | Full Sample  n (%) or M/SD | Obese (6,652)  n (%) or M/SD | Non-Obese (32,837)  n (%) or M/SD | Chi-square/t-test  p-value |
| --- | --- | --- | --- | --- |
| Sex |  |  |  |  |
| Male | 20,319 (51.2) | 3,855 (58.7) | 16,464 (49.8) | <.0001 |
| Female | 19,170 (48.8) | 2,797 (41.3) | 16,373 (50.2) |  |
| Age | 14.6 / 1.7 | 14.7 / 1.6 | 14.6 / 1.7 | <.0001 |
| 12 years | 5,486 (13.9) | 757 (11.2) | 4,729 (14.5) | <.0001 |
| 13 years | 6,313 (15.5) | 1,036 (15.3) | 5,277 (15.6) |  |
| 14 years | 6,651 (17.3) | 1,118 (17.3) | 5,533 (17.3) |  |
| 15 years | 6,954 (17.3) | 2,269 (18.3) | 5,685 (17.1) |  |
| 16 years | 7,123 (18.1) | 1,260 (19.7) | 5,863 (17.8) |  |
| 17 years | 6,692 (17.8) | 1,212 (18.4) | 5,750 (17.7) |  |
| Race/ethnicity |  |  |  |  |
| White | 20,552 (51.8) | 2,888 (43.2) | 17,664 (53.5) | <.0001 |
| Black | 5,557 (14.2) | 1,249 (19.1) | 4,308(13.2) |  |
| Hispanic | 8,841 (23.4) | 1,863 (30.3) | 6,978 (22.1) |  |
| Asian | 1,770 (6.5) | 125 (2.9) | 1,645 (7.2) |  |
| Other | 2,769 (4.1) | 527 (4.5) | 2,242 (4.1) |  |
| Family Income Level |  |  |  |  |
| Less than $20,000 | 6,109 (14.1) | 1,487 (21.3) | 4,532 (12.8) | <.0001 |
| $20,000 - $49,999 | 10,740 (25.8) | 2,253 (32.3) | 8,487 (24.6) |  |
| $50,000 - $74,999 | 5,828 (13.8) | 992 (14.7) | 4,836 (13.6) |  |
| $75,000 or More | 16,902 (46.3) | 1,920 (31.6) | 14,982 (49.1) |  |
| Cigarette smoker |  |  |  |  |
| No | 34,858 (89.3) | 5,682 (86.2) | 29,176 (89.9) | <.0001 |
| Yes | 4,631 (10.7) | 970 (13.8) | 3,661 (10.1) |  |
| Alcohol use | 6.11 / 26.4  (range: 0-364) | 6.22 / 27.0  (range: 0-364) | 6.08 / 26.3  (range: 0-364) | .69 |
| Locality |  |  |  |  |
| Rural | 12,255 (20.0) | 2,327 (24.5) | 9,928 (19.1) | <.0001 |
| Urban | 27,234 (80.0) | 4,325 (75.5) | 22,909 (80.9) |  |

Note. Percentages are based on weighted frequencies.
