# Supplementary material for: Assessing how alcohol use patterns relate to obesity among American adolescents from rural and urban areas: Five years of pooled data
Source: PLoS One. 2024 Jun 27;19(6):e0305638. doi: 10.1371/journal.pone.0305638 (PMC11210796; doi:10.1371/journal.pone.0305638)
Supplement: Attachment — Submitted filename: PLOS_RR_Response to Reviewers_4-26-24.docx [file pone.0305638.s002.docx]

Dear Editors and Reviewers:

Thank you for the thoughtful reviews of our manuscript (PONE-D-24-02462), entitled “Assessing how alcohol use patterns relate to obesity among American adolescents from rural and urban areas: Five years of pooled data.” We appreciate the comments and suggestions and have made the suggested changes to the manuscript. We believe the manuscript is stronger as a result of the review and that it will make a significant contribution to the field.

This manuscript has only been submitted to PLOS ONE and has not been previously published, either in whole or in part, nor have the findings been posted online. As the corresponding author, I have had full access to all aspects of the research and the writing of this manuscript. I take full responsibility for the findings as presented. A revised version with highlighted/tracked changes and a clean version was also submitted.

Below are each of the comments we received with a brief response in red font that indicates the action taken to revise the manuscript.

Editor comments:

You make also note that there is a large literature on the economic losses of alcohol consumption.

RESPONSE: Thank you for pointing us to this literature. We now cite this in the Introduction.

Reviewer Comments:

Reviewer #1: 1. Overall assessment

The research question here is straightforward and the method is nice and simple while sufficing for your goals, making for an unusually digestible scientific paper. The most serious problems with the analysis are the choice of subsample and the construction of not-so-simple effects, but I think both will be easy to address. See below for details.

RESPONSE: Thank you for the comment. We hope the revisions below address your concerns.

2. Title and abstract

2.1. I don't think "Assessing how alcohol use patterns impact obesity…" is a fair title, because "impact" means "affect", and you're not looking at causal evidence. How about "relate to" instead of "impact"?

RESPONSE: We agree and have made this change to the title.

2.2. Mention that the sample comes from the US in the title or abstract. For example, you could change the title to say "among American adolescents from…".

RESPONSE: We add “American” to the title and in the methods section of the abstract.

3. Methods

3.1. "Participants who reported never using alcohol were excluded" — You should probably mention this criterion earlier, while describing the subsample of 11,814, assuming that 11,814 is what you ended up with after applying this criterion.

RESPONSE: Please see response to comment 3.2.

3.2. But the bigger issue is that the exclusion is poorly motivated. Your goal is to examine "the relationship between alcohol use and obesity". By throwing out teetotalers, you're needlessly restricting the range of your alcohol-use variable, preventing you from seeing how alcohol use is related to obesity as it increases from zero to nonzero, and you've probably made the sample a lot smaller, too. You should include the teetotalers.

RESPONSE: The sample size does grow significantly with teetotalers included. To address this comment and comment 3.3 we revised the sample to include the teetotalers and we also created a continuous variable that offers more information by using a different alcohol variable that asks to report total number of days drank in the past 12 months. The variable we created includes 8 categories (used as continuous): 0 = never or not in the past 365 days; 1 = 1–2 times (over the past year); 2 = 3–5 times; 3 = 5 + times but less than monthly; 4 = 1–2 times a month; 5 = 1–2 times a week; 6 = 3–5 times a week; and 7 = nearly daily. The code pairings for the actual values of this variable are 0 = 0; 1 = 1-2; 2 = 3-5; 3 = 6-12; 4 = 13-24; 5 = 25-104; 6 = 105 – 260; and 7 = 260+. This variable has been coded and used in this way by another researcher who also examines drinking frequency. That researcher and I spoke to confirm appropriate replication of the variable. I provide references below and in the manuscript. Revisions have been made to Methods, Results, and Tables.

Waddell JT. Between- and within-group effects of alcohol and cannabis co-use on AUD/CUD in the NSDUH 2002–2019. Drug and Alcohol Dependence. 2021;225:108768. doi:10.1016/j.drugalcdep.2021.108768

Waddell JT. Frequency matters: Relations among alcohol and cannabis co-use frequency and alcohol use disorder symptoms in emerging adults. Experimental and clinical psychopharmacology. 2023;31(4):780. doi:https://doi.org/10.1037/pha0000616

3.3. Having drunk a single alcoholic beverage within the last 30 days seems like a very low bar for "frequent" drinking. That said, I recognize that your coding options are limited by the data, with this being the highest rate respondents could report, and I can't think of a better term. Maybe it's enough to describe earlier in the paper (perhaps in the abstract) what counts as "frequent" and "casual" for this study.

RESPONSE: We agree with this comment; however, we won’t directly address the comment as we made a significant change to the alcohol variable that is explained in comment 3.2. We believe the new coding and description in the Methods section is appropriate and addresses your comment. We revise language to the Variables section and still add new language to the Limitations section.

3.4. "The 'other' race category included Native American/Alaskan Native, Native Hawaiian/Other Pacific Islander, Asian, and more than one race. This was done due to the sample size of these groups." — I bet the result is that the "other" category is almost entirely Asian. (For example, the 2020 Census found that about 7% of the US population is Asian, while your "other" category includes 7.9% of your subsample: https://www2.census.gov/programs-surveys/decennial/2020/data/redistricting-supplementary-tables/redistricting-supplementary-table-01.pdf ) If so, it could make for somewhat more interpretable results to give Asians their own category. The size of the remaining "other" category will be very small, and hence you won't be able to infer much about it, but this avoids the situation where you attribute a specifically Asian effect to a grab bag of races.

RESPONSE: Thank you for the comment. We broke out the race/ethnicity category into 5 categories: Asian, Black, Hispanic, Other (Native American/Alaskan Native, Native Hawaiian/Other Pacific Islander, and more than one race), White.

4. Results

4.1. Self-reported health status would likely be more interpretable in its original five categories ("excellent", "very good", "good", "fair", "poor") than collapsed into two.

RESPONSE: We removed this variable per a comment from Reviewer 2.

4.2. [In a note for Table 2] "Coefficients for controls are not shown" — Why not? They'd be easier to read in this table than where you have them right now, embedded in the prose. And the intercept is in neither the table nor the text.

RESPONSE: We now include all the results in a Table 2. We typically do not include intercepts in Tables. We can add this if you would like to see this in a final revision.

4.3. In "Rural casual drinker vs. Urban frequent drinker" and "Rural frequent drinker vs. Urban casual drinker", you've varying two things at once. So, they aren't simple effects, and they don't really make sense as comparisons. Perhaps you meant to examine "Rural casual drinker vs. Rural frequent drinker" and "Urban casual drinker vs. Urban frequent drinker".

RESPONSE: There was an initial error with the comparisons as you pointed out. Since we are now using a continuous variable for the alcohol variable, we now include a Figure 1 showing predicted probabilities (instead of a table with comparisons), which we believe is the most appropriate choice given the changes to the analysis.

5. Discussion

5.1. "The current study findings suggest there is no difference in the association of having obesity among casual and frequent drinkers." — A failure to obtain significance is not evidence of zero association, and in fact, zero association is an implausible hypothesis. See e.g. https://stats.stackexchange.com/questions/219359

RESPONSE: Thank you for the reminder to make our language more accurate of our findings. The sentence you comment on is no longer relevant in the paper. However, we make sure to revise our wording throughout the discussion to be more appropriate with what our findings represent. See revised Discussion section.

5.2. The section "Limitations" includes the sentences "This was a large nationally representative dataset with more than sufficient sample sizes across groups." and "The current study emphasized urban versus rural differences, an understudied intersection of obesity and alcohol use." Those statements may be true, but neither is stating a limitation or is apparently relevant to the discussion of limitations.

RESPONSE: These sentences have been removed. The Limitations section was further revised to address comments from Reviewer 1 and 2. Please see new Limitations section.

6. Prose issues

6.1. Try to avoid circumlocutions. "Have engaged in alcohol use" is better written "have used alcohol" or "have drunk alcohol". "Being a boy" is better written "being male". "Have obesity" and similar constructions are better written "be obese" ("'0' as not having obesity" can be just "'0' as not obese"), and similarly "adolescents with obesity" is better written "obese adolescents", while "adolescent drinkers from rural areas are more at risk of being an adolescent with obesity" can be just "adolescent drinkers from rural areas are more at risk of obesity". I'd guess that the word "having" in the phrase "a consequence of heavy drinking during adolescence is overweight and having obesity" was just a typo.

RESPONSE: Thank you for the suggestion. We originally wrote this for a journal that preferred this format as it uses person first language, however we understand it is not ideal given the issues you have pointed out. We revised this style of writing for the current version per your suggestion.

6.2. "liquor" — If you mean all alcoholic beverages, it's less ambiguous to say "alcohol".

RESPONSE: This is fixed.

6.3. "there also appears to be mixed findings" — "appears" should be "appear".

RESPONSE: This is fixed.

6.4. "The data from the year 2015 to 2019" — "year" should be "years".

RESPONSE: This is fixed.

6.5. "compared to over $75,00" — Don't forget that last zero.

RESPONSE: This is fixed.

6.6. "an interaction term to examine locality x alcohol use." — Here only the phrase "alcohol use" is italicized, which is presumably a mistake.

RESPONSE: That was an error. This is fixed.

6.7. "To obtain accurate odds ratios (ORs) and 95% confidence intervals (CIs), parameter estimates and their associated upper and lower confidence limits were exponentiated." — I'm not sure what the word "accurate" is doing here. The exponentiation isn't some kind of accuracy correction, just a rescaling transformation, from the log-odds scale to the odds scale.

RESPONSE: This word was removed.

Reviewer #2: In the following study by Vazquez at al., the authors have attempted to analyze the relationship between being an obese adolescent and alcohol intake. Particularly, they have analyzed if there are differences between rural and urban areas and the drinking pattern: casual vs frequent drinking. Although the study is interesting from a public health perspective and the methodology sound, there are some relevant issues to be clarified. The results of the association are a little bit confusing and some important co-variables are missing.

RESPONSE: Thank you for your review. We have made revisions to make the results more interpretable. While there are certain changes we could not make due to data limitations (i.e., variables not available in the dataset), we do attempt to address as many of your concerns as possible. Whatever we could not fully address, we added to limitations. We would like to note that this is one of the few nationally representative datasets in the United States with these pairing of variables and though other smaller datasets may have all the variables you would like to see, we believe the representativeness of this dataset is a strength that gives us some insight into the question at hand. Please see our responses below.

Major comments

The question to classify subjects as casual or frequent drinkers is not direct and could lead to a misclassification. For instance, one adolescent cannot drink alcohol at all but for certain reasons could have drinked alcohol in the previous month. Then, this subject was classified as frequent drinker when in fact, was a casual one.

A more direct question, such as: “do you regularly, at least one a week or twice a month, drink alcohol?” would have been more appropriate.

This has to be acknowledged in the limitation section.

RESPONSE: We acknowledge that this could lead to misclassification. Unfortunately, the question in this dataset was not asked the way you propose. To address this comment we revised the sample to include the non-drinkers and created a continuous variable that offers more information by using a different alcohol variable that asks to report total number of days drank in the past 12 months. The variable we created includes 8 categories (used as continuous): 0 = never or not in the past 365 days; 1 = 1–2 times (over the past year); 2 = 3–5 times; 3 = 5 + times but less than monthly; 4 = 1–2 times a month; 5 = 1–2 times a week; 6 = 3–5 times a week; and 7 = nearly daily. The code pairings for the actual values of this variable are 0 = 0; 1 = 1-2; 2 = 3-5; 3 = 6-12; 4 = 13-24; 5 = 25-104; 6 = 105 – 260; and 7 = 260+. This variable has been coded and used in this way by another researcher who also examines drinking frequency. That researcher and I spoke to confirm appropriate replication of the variable. I provide references below and in the manuscript. We also add a note to limitations regarding the possible misclassification even with the new variable. Revisions have been made to Methods, Results, Tables, and Limitations.

Waddell JT. Between- and within-group effects of alcohol and cannabis co-use on AUD/CUD in the NSDUH 2002–2019. Drug and Alcohol Dependence. 2021;225:108768. doi:10.1016/j.drugalcdep.2021.108768

Waddell JT. Frequency matters: Relations among alcohol and cannabis co-use frequency and alcohol use disorder symptoms in emerging adults. Experimental and clinical psychopharmacology. 2023;31(4):780. doi:https://doi.org/10.1037/pha0000616

Although, I think it could be appropriate to include smoking as independent variable. I wouldn’t talk about the obesity paradox in a population of adolescents due to the fact that is related to the association of overweight or obesity, with less mortality in older people.

RESPONSE: We removed language related to the obesity paradox but keep smoking in as a control.

In the description of the logistic regression model, age appears as a risk factor along with others including black, Hispanic, Poor reported Health and low income, but the OR provided is less than 1. The authors also referred to table 2 but the detailed model with the OR for all the included variables is missing. Also in the interaction terms, it would be necessary to include the OR for each interaction category concerning to the reference category: casual drinkers of urban areas.

RESPONSE: We do not mean to say that those are risk factors. Perhaps our wording read that way. We re-worded the results to make it clear that these were significant but in different directions (i.e., some risk, some protective). We also now include all of the results from the final model in Table 2 and include a Figure to further assess the interaction, which is now continuous by categorical.

I am not keen on including poor reported health as an independent factor for obesity. This variable is very complex and could be significantly related with many other variables. From my point of view, probably, it is the other way around, having obesity being a risk factor for poor reported health. In this sense, I miss variables related with nutritional habits, sedentary behaviour or physical exercise. I do not know if the authors can have access or not to these variables.

RESPONSE: We removed the health variable from the analysis per your suggestion. The data set used for this study does not include variables for nutrition, sedentary behavior, or exercise. We do note this in our Limitations. As mentioned above, this is one of the few nationally representative datasets in the United States with these pairing of variables and though other smaller datasets may have all the variables you would like to see, we believe the representativeness of this dataset gives us insights into the question at hand.

The discussion seems to support that the place of living is more important than the frequency of alcohol consumption due to the fact that frequent drinkers of urban areas have less risk than casual drinkers for rural areas. So the conclusion that adolescents from rural areas who drink may be more at risk of having obesity is at least only partially supported.

RESPONSE: The discussion changed slightly given the changes to the analysis; however, we make sure to be thoughtful with our wording to state that the hypothesis we made is only partially supported. See revied Discussion.

Some limitations should be addressed such as the majority of the studied population belonged to urban areas as compared to rural areas. Maybe some information about the survey and the representativity of the selected population should be added.

RESPONSE: We add new sentences to the Limitations discussing the use of the alcohol variable, inability to include physical activity and nutrition variables, and how the sample may be more representative of urban adolescents. We also include more information to the Data section in the Methods and make it clear where others can find additional information about this dataset.

In my opinion, it would be necessary, as it was made in table 1 for locality, a table comparing the characteristics of obese vs non-obese adolescents. Also it is interesting to highlight with an asterisk or other symbol, those differences statistically significant.

RESPONSE: We include this at the end of this document for your reference. We find that it offers similar information that one would find in the final models. We can include it as a supplementary file if you feel strongly about it but do not believe it should be included in the current version of the manuscript as the main focus is on the rural urban differences, which are not observed from the regression analysis for the rest of the characteristics, excluding obesity. We do however, add the p-values for significant differences across locality in the current version of Table 1.

As participants who reported never using alcohol were excluded, it would be also relevant to include a supplementary table with the characteristics including also those who never reported using alcohol, and maybe comparing frequent, casual drinkers with those who have never drinked.

RESPONSE: Reviewer one wanted to see this group added back into the analysis. We have done that. This also addresses your comment. Please see new analysis, results, and tables.

Minor comments

In control variables it is mentioned smoking but does not appear as a variable of adjustment in table 2.

RESPONSE: This was a typo. Smoking was included as a control for the analysis.

There is a parenthesis missing in Table 1.

RESPONSE: Thank you. We fixed this.

The introduction section is somewhat long and redundant concerning the knowledge gap and the purpose of the study.

RESPONSE: We made some revisions throughout the Introduction to help cut sentences and synthesize to reduce redundancy. Please see Introduction for changes.

The article does not contain figures, it would be interesting to plot the results of the interaction.

RESPONSE: We now include a Figure 1 with the plot of predicted probabilities for the interaction results.

**Supplementary Table 1. Descriptive characteristics of the study sample by Obese vs Not Obese**

|  | Full Sample  n (%) or M/SD | Obese (6,652)  n (%) or M/SD | Not obese (32,837)  n (%) or M/SD | Chi-square/t-test  p-value |
| --- | --- | --- | --- | --- |
| Sex |  |  |  |  |
| Male | 20,319 (51.2) | 3,855 (58.7) | 16,464 (49.8) | <.0001 |
| Female | 19,170 (48.8) | 2,797 (41.3) | 16,373 (50.2) |  |
| Age | 14.6 / 1.7 | 14.7 / 1.6 | 14.6 / 1.7 | <.0001 |
| 12 years | 5,486 (13.9) | 757 (11.2) | 4,729 (14.5) | <.0001 |
| 13 years | 6,313 (15.5) | 1,036 (15.3) | 5,277 (15.6) |  |
| 14 years | 6,651 (17.3) | 1,118 (17.3) | 5,533 (17.3) |  |
| 15 years | 6,954 (17.3) | 2,269 (18.3) | 5,685 (17.1) |  |
| 16 years | 7,123 (18.1) | 1,260 (19.7) | 5,863 (17.8) |  |
| 17 years | 6,692 (17.8) | 1,212 (18.4) | 5,750 (17.7) |  |
| Race/ethnicity |  |  |  |  |
| White | 20,552 (51.8) | 2,888 (43.2) | 17,664 (53.5) | <.0001 |
| Black | 5,557 (14.2) | 1,249 (19.1) | 4,308(13.2) |  |
| Hispanic | 8,841 (23.4) | 1,863 (30.3) | 6,978 (22.1) |  |
| Asian | 1,770 (6.5) | 125 (2.9) | 1,645 (7.2) |  |
| Other | 2,769 (4.1) | 527 (4.5) | 2,242 (4.1) |  |
| Family Income Level |  |  |  |  |
| Less than $20,000 | 6,109 (14.1) | 1,487 (21.3) | 4,532 (12.8) | <.0001 |
| $20,000 - $49,999 | 10,740 (25.8) | 2,253 (32.3) | 8,487 (24.6) |  |
| $50,000 - $74,999 | 5,828 (13.8) | 992 (14.7) | 4,836 (13.6) |  |
| $75,000 or More | 16,902 (46.3) | 1,920 (31.6) | 14,982 (49.1) |  |
| Cigarette smoker |  |  |  |  |
| No | 34,858 (89.3) | 5,682 (86.2) | 29,176 (89.9) | <.0001 |
| Yes | 4,631 (10.7) | 970 (13.8) | 3,661 (10.1) |  |
| Alcohol use | 0.66 / 1.5 | 0.66 / 1.5 | 0.66 / 1.5 | .99 |
| Never or not in the past 12 months | 30,600 (77.9) | 5,132 (76.9) | 25,468 (78.1) | .47 |
| 1-2 over the past 12 months | 2,385 (6.0) | 428 (6.9) | 1,957 (5.8) |  |
| 5-5 times | 1,868 (4.7) | 312 (4.9) | 1,556 (4.7) |  |
| 5+ times but less than monthly | 1,435 (3.6) | 251 (3.5) | 1,184 (3.6) |  |
| 1-2 times a month | 878 (2.3) | 151 (2.5) | 727 (2.2) |  |
| 1-2 times a week | 1,816 (4.4) | 284 (4.1) | 1,532 (4.5) |  |
| 3-5 times a week | 426 (1.0) | 82 (1.2) | 344 (1.0) |  |
| Nearly daily | 81 (0.2) | 12 (0.1) | 69 (0.2) |  |
| Locality |  |  |  |  |
| Rural | 12,255 (20.0) | 2,327 (24.5) | 9,928 (19.1) | <.0001 |
| Urban | 27,234 (80.0) | 4,325 (75.5) | 22,909 (80.9) |  |

Note. Percentages are based on weighted frequencies.
