# Supplementary material for: Assessing how alcohol use patterns relate to obesity among American adolescents from rural and urban areas: Five years of pooled data
Source: PLoS One. 2024 Jun 27;19(6):e0305638. doi: 10.1371/journal.pone.0305638 (PMC11210796; doi:10.1371/journal.pone.0305638)
Supplement: Attachment — Submitted filename: PLOS_RR_Response to Reviewers_5-14-24.docx [file pone.0305638.s003.docx]

Dear Editors and Reviewers:

Thank you for the thoughtful reviews of our manuscript (PONE-D-24-02462), entitled “Assessing how alcohol use patterns relate to obesity among American adolescents from rural and urban areas: Five years of pooled data.” We appreciate the comments and suggestions and have made the suggested changes to the manuscript. We believe the manuscript is stronger as a result of the review and that it will make a significant contribution to the field.

This manuscript has only been submitted to PLOS ONE and has not been previously published, either in whole or in part, nor have the findings been posted online. As the corresponding author, I have had full access to all aspects of the research and the writing of this manuscript. I take full responsibility for the findings as presented. A revised version with highlighted/tracked changes and a clean version was also submitted.

Below are each of the comments we received with a brief response in red font that indicates the action taken to revise the manuscript.

Editor comments:

Please review your reference list to ensure that it is complete and correct. If you have cited papers that have been retracted, please include the rationale for doing so in the manuscript text, or remove these references and replace them with relevant current references. Any changes to the reference list should be mentioned in the rebuttal letter that accompanies your revised manuscript. If you need to cite a retracted article, indicate the article’s retracted status in the References list and also include a citation and full reference for the retraction notice.

RESPONSE: We are not sure if this is an automated comment from the system, but we checked our references and made some minor updates to capitalization of letters. Overall, we feel it is complete and correct. We did not identify any articles that were retracted but please let us know if that is the case and we will happily replace it.

Reviewer Comments:

Reviewer #2: In my view, the authors have effectively addressed all of my previous comments, resulting in improvements to the manuscript. However, I am concerned about the apparent inverse relationship observed between alcohol intake and obesity risk when alcohol intake is treated as a continuous variable. Additionally, the decision to categorize the alcohol intake variable and subsequently treat these categories as continuous is questionable. A potentially clearer approach might involve using the number of drinking days, assigning a value of 0 for those who have not consumed alcohol in the past 365 days or have never drunk alcohol.

Furthermore, in the logistic regression analysis, the odds ratio (OR) is below 1, though it is not statistically significant. Given these factors, I acknowledge that the impact of alcohol on obesity risk might differ between rural and urban areas. However, it remains unclear whether alcohol intake is a risk factor for obesity in this specific population, or conversely, if it might be protective.

RESPONSE: Thank you for your review. We understand the main concerns. We re-ran the analysis using number of drinking days (0 to 365 days) as suggested. The change to the analysis resulted in some minor changes to the findings which relate to your follow up comment. The inverse was not distinguishable. The main effect and predicted probability results contribute to the inconsistent findings in the literature. Figure 1 shows that there are differences between rural and urban adolescents at low levels of drinking. Further it also appears that this may change at higher levels of drinking; however, this change is not statistically distinguishable. Thus, we do not go as far as saying that drinking is a protective factor because and it would not be prudent to suggest adolescents should drink alcohol to prevent obesity. We are transparent in what the results are but point to future research needing to understand what mechanisms are in play that might possibly mimic a protective factor against obesity for high frequency adolescent rural drinkers (i.e., those who are drinking about 125 days or more per year). Revisions are made to Methods, Results, Tables/Figures, and Discussion.

Regarding the presentation of data, I recommend including the table that compares obese versus non-obese individuals as a supplementary table. Also, I am unsure if "Non-Obese" or "Not Obese," as currently used in the manuscript, is preferable. "Non-Obese" might be more standard and clearer.

RESPONSE: This table is now included as a supplementary file, and we also make the wording change to non-obese throughout the manuscript.

**Supplementary Table 1. Descriptive characteristics of the study sample by Obese vs Non-Obese, National Survey on Drug Use and Health, Years 2015-2019, Adolescent sample (age 12-17; n = 39,489)**

|  | Full Sample  n (%) or M/SD | Obese (6,652)  n (%) or M/SD | Non-Obese (32,837)  n (%) or M/SD | Chi-square/t-test  p-value |
| --- | --- | --- | --- | --- |
| Sex |  |  |  |  |
| Male | 20,319 (51.2) | 3,855 (58.7) | 16,464 (49.8) | <.0001 |
| Female | 19,170 (48.8) | 2,797 (41.3) | 16,373 (50.2) |  |
| Age | 14.6 / 1.7 | 14.7 / 1.6 | 14.6 / 1.7 | <.0001 |
| 12 years | 5,486 (13.9) | 757 (11.2) | 4,729 (14.5) | <.0001 |
| 13 years | 6,313 (15.5) | 1,036 (15.3) | 5,277 (15.6) |  |
| 14 years | 6,651 (17.3) | 1,118 (17.3) | 5,533 (17.3) |  |
| 15 years | 6,954 (17.3) | 2,269 (18.3) | 5,685 (17.1) |  |
| 16 years | 7,123 (18.1) | 1,260 (19.7) | 5,863 (17.8) |  |
| 17 years | 6,692 (17.8) | 1,212 (18.4) | 5,750 (17.7) |  |
| Race/ethnicity |  |  |  |  |
| White | 20,552 (51.8) | 2,888 (43.2) | 17,664 (53.5) | <.0001 |
| Black | 5,557 (14.2) | 1,249 (19.1) | 4,308(13.2) |  |
| Hispanic | 8,841 (23.4) | 1,863 (30.3) | 6,978 (22.1) |  |
| Asian | 1,770 (6.5) | 125 (2.9) | 1,645 (7.2) |  |
| Other | 2,769 (4.1) | 527 (4.5) | 2,242 (4.1) |  |
| Family Income Level |  |  |  |  |
| Less than $20,000 | 6,109 (14.1) | 1,487 (21.3) | 4,532 (12.8) | <.0001 |
| $20,000 - $49,999 | 10,740 (25.8) | 2,253 (32.3) | 8,487 (24.6) |  |
| $50,000 - $74,999 | 5,828 (13.8) | 992 (14.7) | 4,836 (13.6) |  |
| $75,000 or More | 16,902 (46.3) | 1,920 (31.6) | 14,982 (49.1) |  |
| Cigarette smoker |  |  |  |  |
| No | 34,858 (89.3) | 5,682 (86.2) | 29,176 (89.9) | <.0001 |
| Yes | 4,631 (10.7) | 970 (13.8) | 3,661 (10.1) |  |
| Alcohol use | 6.11 / 26.4  (range: 0-364) | 6.22 / 27.0  (range: 0-364) | 6.08 / 26.3  (range: 0-364) | .69 |
| Locality |  |  |  |  |
| Rural | 12,255 (20.0) | 2,327 (24.5) | 9,928 (19.1) | <.0001 |
| Urban | 27,234 (80.0) | 4,325 (75.5) | 22,909 (80.9) |  |

Note. Percentages are based on weighted frequencies.
